# Unmasking the Hidden Burden: A Delayed Diagnosis of Leprosy Patients With Grade 2 Disability and Its Effects on the Healthcare System in Bangladesh

**DOI:** 10.7759/cureus.58708

**Published:** 2024-04-22

**Authors:** Susmita Sinha, Md. Ahsanul Haq, Rahnuma Ahmad, Suman Banik, Santosh Kumar, Mainul Haque

**Affiliations:** 1 Physiology, Khulna City Medical College and Hospital, Khulna, BGD; 2 Biostatistics, icddr,b, Dhaka, BGD; 3 Physiology, Medical College for Women and Hospital, Dhaka, BGD; 4 Administration, Directorate General of Health Services (DGHS), Dhaka, BGD; 5 Periodontology and Implantology, Karnavati School of Dentistry, Karnavati University, Gandhinagar, IND; 6 Therapeutics, Karnavati Scientific Research Center (KSRC), School of Dentistry, Karnavati University, Gandhinagar, IND; 7 Pharmacology and Therapeutics, National Defence University of Malaysia, Kuala Lumpur, MYS

**Keywords:** mycobacterium leprae, neglected tropical disease, lower socioeconomic group, grade 2 disability, socioeconomic status, social stigma, delayed diagnosis, leprosy, g2d, hansen’s disease

## Abstract

Introduction

Leprosy remains a significant cause of preventable disability worldwide. Early diagnosis and treatment of leprosy are critical not only to stop its spread but also to prevent physical and social complications and reduce the disease burden.

Objectives

The study aims to evaluate the factors that lead to a delayed leprosy diagnosis.

Methods

This study was conducted in the outpatient departments of Leprosy Control Institute and Hospital, Dhaka, Bangladesh, and at Medical College for Women and Hospital, Dhaka, Bangladesh, from March 2023 to June 2023. A total number of 252 male (148) and female (104) patients were selected with any sign of leprosy, including disability, age ranging from 15 to 74 years. Data was collected in a pre-designed structured questionnaire by the researchers. To assess the risk of independent exposures of Grade 2 leprosy disabilities, we used a logistic regression model. A chi-square test showed the association between significant effects and leprosy disabilities. A p-value of 0.05 was considered as significant. For statistical analysis, STATA version 15 (StataCorp LLC, College Station, Texas, USA) was used.

Results

The study participants exhibited a higher percentage of disability, with a rate of 25.8% for Grade 2 disabilities. In addition to this, males represented a more considerable proportion, 58.7%, than females among leprosy and disability patients across all levels of disability. In our study, lack of money and painless symptoms showed a significant association (p<0.001) with Grade 2 disability.

Conclusion

The study reveals that Grade 2 disabilities are more common in males and are particularly prevalent in lower socioeconomic groups.

## Introduction

Hansen's disease, often known as leprosy, is known to induce deformity and impairment, and major repercussions may result from a delay in the diagnosis of leprosy patients [1]. Peripheral nerves are affected most, followed by skin, mucous membranes, and other bodily tissues such as the eyes, muscles, bones, testes, and internal organs [2]. The main methods for controlling leprosy are early detection and prompt treatment of all newly identified cases with multidrug therapy (MDT), as advised by the World Health Organization (WHO) [3]. Bangladesh met the national goal of eliminating leprosy as a public health hazard (fewer than one case per 10,000 people) in 1998, two years earlier than the WHO's goal of eliminating leprosy by 2000 [4]. The 2016-2020 global leprosy plan assured a more remarkable dedication to further reducing the detrimental effects of the disease and preventing leprosy-affected children from developing lifelong disabilities [5]. Clinical examinations of individuals and careful history collection are the two essential components of leprosy diagnosis [6]. Leprosy typically develops slowly, making it challenging to spot a case early in the illness. Despite the availability of effective treatment options, delayed diagnosis of leprosy is still expected in many settings, which can lead to the development of a Grade 2 disability (G2D). This disability can have significant physical, psychological, and economic consequences for individuals and society [7]. Leprosy facilities were integrated into the entire healthcare network to provide timely evaluation, management, and prompt therapy when leprosy became known to be no longer a health concern in Bangladesh [8]. Despite improved efforts, leprosy is believed to be diagnosed too late, according to reports and research. Leprosy is a public health issue because of the difficulties it creates, and the costs connected with its management [9]. Even though the literature addresses several causes of disability, understanding the magnitude of individual issues, whether unaccompanied or in combination, permits health experts, supervisors, and investigators to develop preventive and therapeutic methods and helps leprosy programmers prioritize interventions [10]. It is crucial to identify program barriers and develop practical solutions to encourage early identification and avoid impairment by evaluating the various causes of delays in seeking care and receiving a leprosy diagnosis. Leprosy must be identified and treated promptly to minimize the chance of developing disabilities and related problems. Despite efforts to eliminate leprosy, it remains a significant cause of preventable disability worldwide.

Several studies conducted in different countries have identified various factors, including sociodemographic characteristics, healthcare-seeking behavior, and health system performance, contributing to the delay in leprosy diagnosis [11]. Most new cases are concentrated in India, Brazil, Indonesia, Bangladesh, and Ethiopia [12]. Within these countries, leprosy tends to be unevenly distributed, with certain regions experiencing higher prevalence [13]. Generally, communities in resource-limited settings are disproportionately affected by leprosy [14]. Bangladesh has been considered a high-endemic country for leprosy for many decades, with significant clusters found in the southeast (Cox's Bazar), central regions (around the capital city of Dhaka), and northwest (around Rangpur, Nilphamari, and Saidpur city) [15]. In 2018 and 2019, approximately four thousand new cases of leprosy were detected each year [16]. Therefore, this study aims to investigate the consequences of delayed diagnosis of leprosy on the development of G2D, which is a critical issue in the management of this disease.

Objectives of the study

This study aims to Identify the factors contributing to delayed diagnosis of leprosy in the study population.

## Materials and methods

Study design

A mixed method (quantitative and qualitative) will be applied to conduct this study. In the quantitative part, a cross-sectional design will be applied.

Study place and period

The study was conducted in the outpatient departments of Leprosy Control Institute and Hospital, Dhaka, Bangladesh, and at Medical College for Women and Hospital, Dhaka, Bangladesh, from March 2023 to June 2023.

Study population

For the quantitative and qualitative studies, patients with different grades of leprosy, key personnel related to leprosy services, and G2D leprosy patients were enrolled.

Selection criteria

The inclusion criteria for this study include individuals aged 18 to 75 years exhibiting any sign of leprosy, including disability, who provide informed consent to participate. Both male and female participants meeting these criteria are eligible for inclusion. Exclusion criteria encompass individuals under 18 years of age, patients without any signs of leprosy, and those who have declined to participate in the study.

Sampling technique

The sampling technique employed for this study is purposive sampling.

Data collection

The data will be collected through a structured questionnaire administered to the patients who meet the inclusion criteria. The questionnaire includes both closed-ended and open-ended questions. Open-ended and direct conversations were employed to collect qualitative data, whereas closed-ended questionnaires were used to gather quantitative data.

Ethical approval

This research obtained ethical approval from the Institutional Review Board of Medical College for Women and Hospital, Dhaka, Bangladesh, with Reference No.: MCWH/ethical committee/2023, Dated: 31/05/2023. Furthermore, the researchers gave the patients a detailed explanation of their research goals and plans for future publications. Written informed consent was obtained from every research participant before the researchers conducted any intervention.

Impact of this research work

This study will contribute to a better understanding of the consequences of delayed diagnosis of leprosy, particularly in the context of G2D. The findings will inform the development of interventions to advance the primary exposure and treatment of leprosy and reduce the burden of disability resulting from this disease. This study will also provide insights into the economic and psychosocial impact of delayed diagnosis and disability, which can inform policy decisions and resource allocation in the healthcare system.

Statistical analysis plan

To determine the essential demographic characteristics, we performed a descriptive analysis. We used a logistic regression model to assess the risk of independent exposures to Grade 2 leprosy disabilities. We also used the chi-square test to see the association between significant effects and leprosy disabilities. A p-value of 0.05 was considered important. For statistical analysis, we used STATA version 15 (StataCorp LLC, College Station, Texas, USA).

## Results

The participants' mean age was 40.2 years with standard deviation (SD)=14.1, and the median age was 38.0 years, ranging from 15 to 74 years. In terms of gender distribution, 58.7% (148/252) were male and 41.3% (104/252) were female. Education-wise, the majority had secondary education (48.0%, 121/252), followed by primary education (32.5%, 82/252), while 15.9% (40/252) had higher secondary education and 3.60% (9/252) had no education. Socio-demographic status varied, with the highest proportion in the upper middle class (30.6%, 77/252) and the lowest in the upper class (6.00%, 15/252). Regarding disability grade, Grade 1 had the highest representation (38.1%, 96/252), followed closely by Grade 0 (36.1%, 91/252), with Grade 2 constituting 25.8% (65/252) (Table 1).

**Table 1:** Demographic Characteristics of Study Participants Data was presented as mean±SD or number with percent (%) in the parenthesis.

Variables	Observations (n=252)
Age, years	40.2±14.1
Age, median (min, max)	38.0(15.0, 74.0)
Sex	
Male	148(58.7%)
Female	104(41.3%)
Education	
No education	9(3.60%)
Primary education	82(32.5%)
Secondary education	121(48.0%)
Higher secondary education	40(15.9%)
Socio-demographic status	
Lower class	52(20.6%)
Lower middle class	35(13.9%)
Middle class	73(29.0%)
Upper middle class	77(30.6%)
Upper class	15(6.00%)
Disability grade	
Grade 0	91(36.1%)
Grade 1	96(38.1%)
Grade 2	65(25.8%)

Table 2 illustrates the distribution of leprosy disability grades across various independent exposures such as sex, education level, and socio-demographic status. Within each disability grade category (Grade 0, Grade 1, and Grade 2), the table provides the number of participants (n) alongside the corresponding percentages for each exposure variable. For instance, within Grade 0, males constituted 35.1%, while females constituted 37.5%. Education-wise, those with secondary education were predominant across all disability grades, ranging from 19.8% to 46.3%. The socio-demographic status also exhibited variations, with lower classes showing higher proportions in Grade 2, while the upper class had higher proportions in Grade 0. The data is presented in a tabular format for clear comparison and analysis.

**Table 2:** Distribution of Leprosy Disability Grades Between Independent Exposures Data was presented as the number with percentage in the parenthesis.

Variables	Grade 0 (n=91)	Grade1 (n=96)	Grade2 (n=65)
Sex			
Male	52(35.1%)	51(34.5%)	45(30.4%)
Female	39(37.5%)	45(43.3%)	20(19.2%)
Education			
No education	1(11.1%)	2(22.2%)	6(66.6%)
Primary education	22(26.8%)	35(42.7%)	25(30.5%)
Secondary education	56(46.3%)	41(33.9%)	24(19.8%)
Higher secondary education	12(30.0%)	18(45.0%)	10(25.0%)
Socio-demographic status			
Lower class	9(17.3%)	9(17.3%)	34(65.4%)
Lower middle class	11(31.4%)	11(31.4%)	13(37.1%)
Middle class	23(31.5%)	40(54.8%)	10(13.7%)
Upper middle class	39(50.6%)	31(40.3%)	7(9.10%)
Upper class	9(60.0%)	5(33.3%)	1(6.70%)

While assessing the associated risk factors of higher-grade leprosy disabilities compared to Grade 0. No risk factors for sex or education were observed. Whereas middle class, upper middle class, and upper class had a lower risk of Grade 2 by 0.12 (95% confidence interval (CI)=0.04, 0.37, p<0.001), 0.05 (95% CI=0.02, 0.16, p<0.001) and 0.04 (95% CI=0.004, 0.39, p=0.006) times respectively (Table 3).

**Table 3:** Associated Risk Factors of Having Grade 2 Leprosy Disabilities A multinomial logistic regression model was used to estimate the p-value. A p-value of <0.05 is considered significant.

Variables	Grade 0 (n=91)	Grade 1 (n=96)		Grade 2 (n=65)	
		OR(95% CI)	p	OR(95% CI)	p
Sex					
Male	1 (Ref)	1 (Ref)		1 (Ref)	
Female		1.17(0.65, 2.16)	0.581	0.69(0.31, 1.51)	0.350
Education					
No education	1 (Ref)	1 (Ref)		1 (Ref)	
Primary education		0.84(0.07, 10.2)	0.869	0.25(0.02, 2.61)	0.254
Secondary education		0.41(0.03, 5.00)	0.483	0.12(0.01, 1.23)	0.075
Higher secondary education		0.83(0.06, 11.6)	0.890	0.31(0.02, 4.18)	0.378
Socio-demographic status					
Lower class	1 (Ref)	1 (Ref)		1 (Ref)	
Lower middle class		1.08(0.30, 3.86)	0.904	0.39(0.13, 1.22)	0.105
Middle class		1.68(0.57, 4.95)	0.343	0.12(0.04, 0.37)	<0.001
Upper middle class		0.78(0.27, 2.25)	0.646	0.05(0.02, 0.16)	<0.001
Upper class		0.64(0.15, 2.83)	0.557	0.04(0.004, 0.39)	0.006

The analysis indicates the association between disability grade and various factors such as visiting local physicians, visiting health institutes, fear of stigma, lack of money, presence of painless symptoms, and duration of delay in seeking medical attention. For instance, 100% of participants who did not visit local physicians were classified as Grade 2, compared to 28.6% of those who did. Similarly, lack of money showed a significant association with Grade 2 leprosy disability, where 88.7% of those reporting financial constraints were categorized as Grade 2 (Table 4).

**Table 4:** Major Factors That Are Associated With Disability Grade 2 Leprosy Data was presented as the number with percentage in the parenthesis. The chi-square test was used to estimate the p-value. A p-value of <0.05 is considered significant.

Variables	Grade 1	Grade 2	p-value
Visiting local physician			
Yes	0	25(100%)	0.003
No	16(28.6%)	40(71.4%)	
Visiting health institute			
Yes	8(18.6%)	35(81.4%)	0.782
No	8(21.1%)	30(78.9%)	
Fear stigma			
Yes	16(21.9%)	57(78.1%)	0.451
No	1(11.1%)	8(88.9%)	
Lack of money			
Yes	6(11.3%)	47(88.7%)	0.004
No	11(37.9%)	18(62.1%)	
Painless symptoms			
Yes	17(32.7%)	35(67.3%)	<0.001
No	0	30(100%)	
Duration of delay			
<12 months	9(23.7%)	29(76.3%)	0.540
>12 months	8(18.2%)	36(81.8%)	

## Discussion

The cause and transmission of leprosy

*Mycobacterium leprae *infection is the primary cause of the chronic granulomatous illness known as leprosy which is also denoted as Hansen's disease since this organism was discovered to cause the disease in 1873 by Norwegian scientist G. Armauer Hansen [17]. A gram-positive, rod-shaped, acid-fast bacillus known as *M. leprae* functions as an essential intracellular parasite. It can be found in skin smears or biopsy specimens from infected persons [18]. Humans are considered the primary host and reservoir of leprosy bacilli. However, the exact mechanism of transmission remains unclear. It is commonly believed that continuous close interaction between people with multibacillary (MB) leprosy will spread the disease primarily by droplets. As a result, compared to families without cases, families with documented cases are likely to have a greater prevalence of leprosy [19].

Epidemic patterns and contagiousness of leprosy

**Figure 1:**
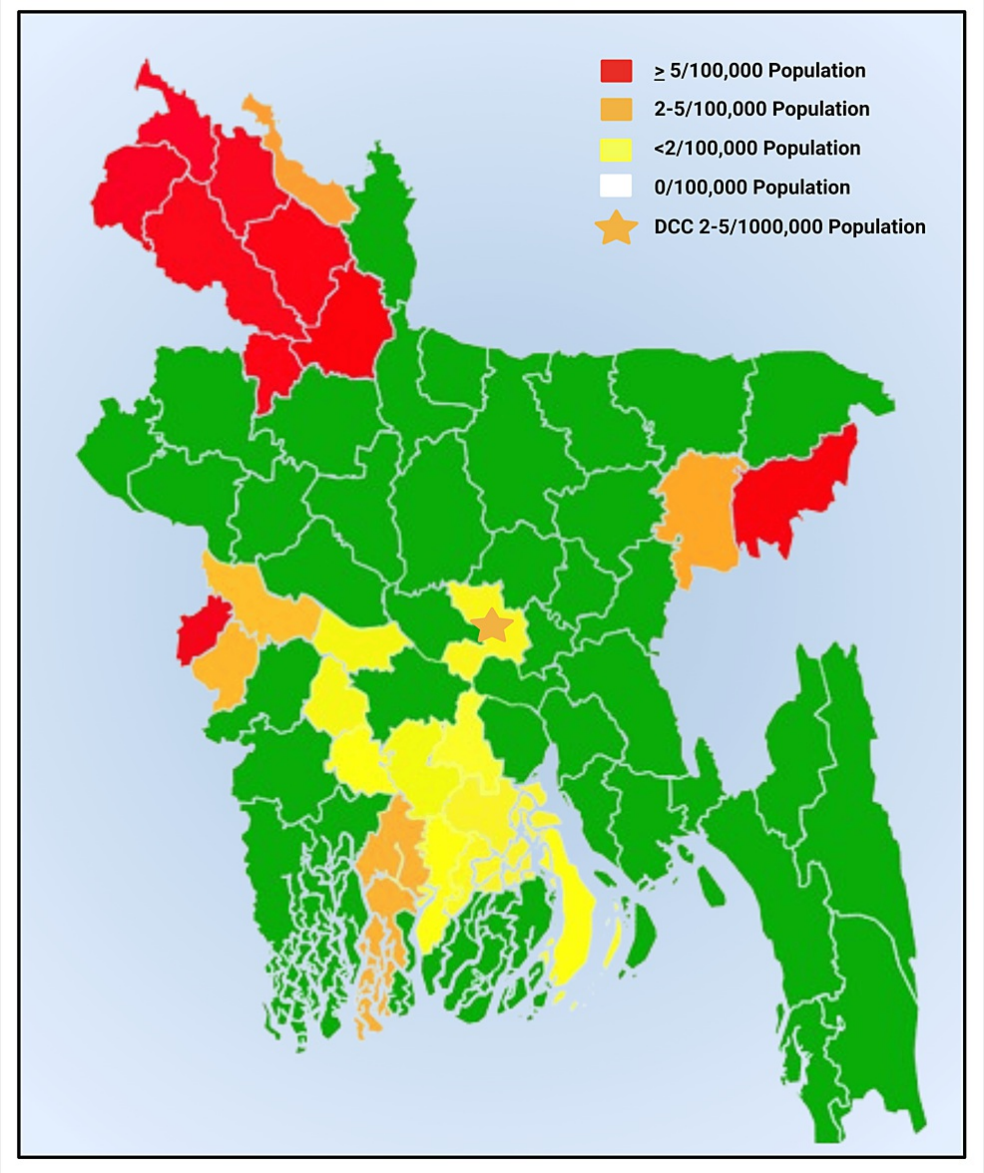
Schematic Diagram Showing the New Case Detection 2022 by the National Leprosy Program, Bangladesh, and Reported by the World Health Organization References: Bangladesh [21] and World Health Organization [22]. This figure has been drawn with the premium version of BioRender [23]; Agreement License number YK26ODVE4G; DCC: detected case count Image credit: Susmita Sinha

Most leprosy cases have been in tropical and subtropical areas, while it happened in northern temperate regions. Consequently, there are no discernible seasonal or geographic differences in the prevalence of leprosy [20]. According to WHO and the National Leprosy Program (NLP) Bangladesh, in 2022, the registered prevalence of leprosy was 2439 (Figure 1) [21,22].

Different categories of leprosy-inflicted disability

The different categories of leprosy-inflicted disability are illustrated in Table 5.

**Table 5:** Various Categories of Disability Due to Leprosy Adapted from Rathod et al. [24] and Brandsma et al. [25].

The WHO Leprosy Disability Grading System
Hands and feet
Grade 0: No anesthesia, no visible deformity or damage.
Grade 1: Anesthesia is present, but no visible deformity or damage.
Grade 2: Visible deformity or damage present.
Eyes
Grade 0: No eye problem due to leprosy; no evidence of visual loss.
Grade 1: Eye problems due to leprosy present, but vision not severely affected as a result (vision: 6/60 or better; can count fingers at 6 meters).
Grade 2 Severe visual impairment (vision worse than 6/60); inability to count fingers at 6 meters); also includes lagophthalmos, iridocyclitis, and corneal opacities.

**Figure 2:**
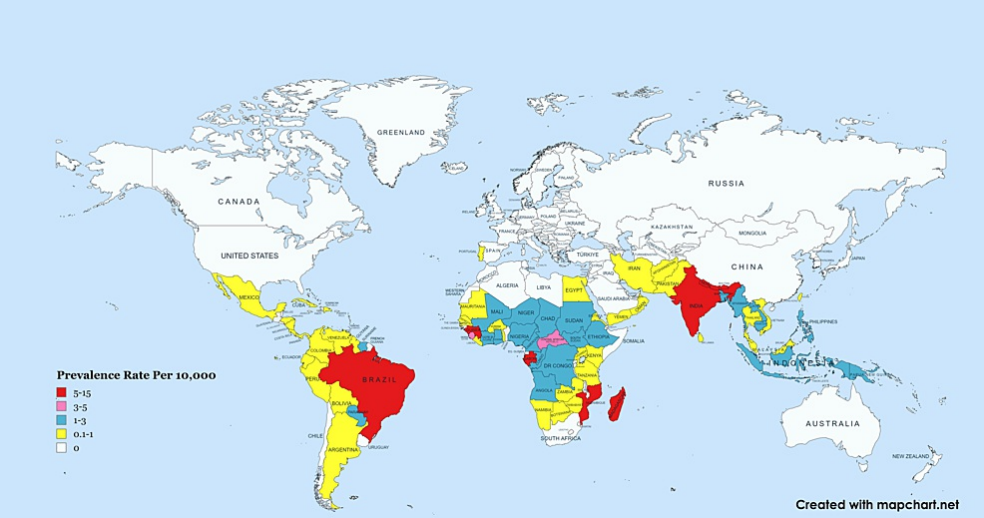
World Map Showing the Detection of New Cases in 2016 This figure is created with a free version of mapchart.net [29].

Previously, in 2016, 214,783 new cases were recorded by WHO from 143 different countries worldwide. This means that 2.9 new cases were detected globally for every 100,000 people (Figure 2) [26]. In addition, leprosy is frequently diagnosed among lower socioeconomic populations of developing countries. Leprosy is believed to be caused by overcrowded living environments, inadequate medical care, nutritional deficiency, and poor sanitary facilities [27]. Nevertheless, there is not much available published data that supports the associations. Leprosy vulnerability and manifestation in an individual have been suggested to be impacted by a genetic flaw, perhaps linked to immune response genes. *M. leprae* infection can result in paucibacillary illness, which is marked by firm granulomas and a Th1 T-cell (cell-mediated) response, or MB disease, which is marked by cellular infiltrates that are ill-organized and Th2 (humoral response) cytokines [28].

Consequences of delayed diagnosis in leprosy-affected patients

G2D, also known as Disability Grade 2 (DG2), represents a significant level of disfigurement and functional impairment caused by leprosy [30]. In this study, G2D is operationalized as visible physical deformities or disabilities resulting from leprosy, such as severe sensory loss, motor weakness, limb deformities, or facial disfigurement. In addition, delayed diagnosis refers to the time elapsed from the onset of symptoms related to leprosy to the formal diagnosis of the disease [31]. It can be quantified as the duration in months or years between the first appearance of symptoms and the confirmation of leprosy through clinical examination and laboratory testing [32].

The study participants exhibited a higher percentage of disability, with a rate of 25.8% for G2Ds. This is significantly higher than the global average of 21.8 per 1,000,000 population and noticeably higher than the WHO's stated rate of 1.09 per 1,000,000 population among newly documented G2D national cases from 2022 [22,33]. This could explain any differences since the patients were mainly from a specialty leprosy hospital, which often serves more complicated or severe patients who have previously received inadequate care elsewhere. In addition to this, males represented a more significant proportion, 58.7%, than females among leprosy and disability patients across all levels of disability. This finding is consistent with a study conducted in Eastern Ethiopia where 80% of males among the study subjects were affected by leprosy [34]. In G2D patients, a higher level of education was linked to a decreased risk of detection delay; this association was not statistically significant. However, another study found a correlation between lower levels of schooling and a higher prevalence of disabilities in Grades 1 or 2 [35]. The primary requirements in lowering disabilities among leprosy patients are health education regarding leprosy, knowledge about the provision of accessible and no-cost healthcare facilities, and follow-up of long-duration courses of multidrug treatment.

Again, lower socioeconomic groups had a higher percentage of people with disabilities, according to a substantial link between socioeconomic position and the grade of disability [36]. While assessing the associated risk factors of higher-grade leprosy disabilities compared to Grade 0 regarding the socio-economic status, middle class, upper middle class, and upper class had a lower risk of G2Ds by 0.12 (95% CI=0.04, 0.37, p<0.001), 0.05 (95% CI=0.02, 0.16, p<0.001) and 0.04 (95% CI=0.004, 0.39, p=0.006) times respectively. According to an epidemiological survey, most patients come from lower socioeconomic backgrounds [37]. The higher prevalence of disease observed in the lower socioeconomic group could be attributed to lifestyle habits, increased environmental exposure to disease, and population growth. Similarly, there are several factors, such as visiting local physicians, visiting health institutes, fear of stigma, lack of money, presence of painless symptoms, and duration of delay in seeking medical attention, contributing to the development of G2Ds. In our study, lack of money and painless symptoms showed a significant association with G2D. Again, this study found that many individuals waited long to seek medical attention for non-alarming, painless symptoms.

A study in Brazil found that approximately 45% of patients did not receive a diagnosis sooner since they weren't aware of the seriousness of their medical conditions [11]. Similar findings were seen in another Indian study that attributed delayed presentation to a lack of knowledge about leprosy symptoms [9]. Additionally, our analysis shows that a fear of stigma is a reliable pointer to a case detection delay. A review found that the stigma associated with leprosy led to delayed discovery among female patients [38]. Therefore, raising awareness about leprosy among community and health personnel is crucial for early detection and constructive views regarding the disease.

Limitations of the study

The study included a limited sample size or focused on specific regions, which could affect the generalizability of the findings to larger populations or different geographical areas. The study also relied on self-reported data, which can be subject to recall bias or misinterpretation.

## Conclusions

MDT, a combination of antibiotics, is a curative treatment for leprosy. Worldwide, there is no cost associated with this treatment. Leprosy can cause significant consequences if left untreated. Fear of stigma and painless manifestations, which are commonly misinterpreted as non-alarming at the beginning of the disease, have been connected to a delay in case diagnosis. We can increase awareness of the illness and patronage a solution to the discrimination and stigma associated with it. Leprosy-related disability affects millions of people worldwide, especially in Asia, South America, and Africa. Moreover, leprosy is a preventable and curable disease, and suffering from leprosy is needless indeed.

## Figures and Tables

**Figure 3:**
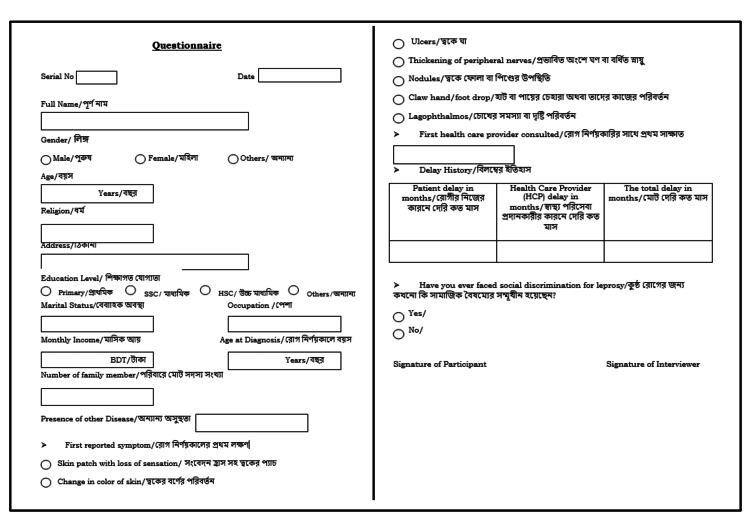
The Instrument of the Study
